# Natural fiber filaments transforming the future of sustainable 3D printing

**DOI:** 10.1016/j.mex.2025.103385

**Published:** 2025-05-22

**Authors:** Senthil Maharaj Kennedy, Lenin Anselm Wilson, Joemax Agu M, Rajeev D, Jeen Robert RB, Balamurugan S

**Affiliations:** a Department of Mechanical Engineering, AAA College of Engineering and Technology, Sivakasi, Tamilnadu 626005, India; b Mechanical Section, Engineering Department, University of Technology and Applied Sciences, Shinas Sultanate of Oman – 324, India; c Faculty of Mechanical Engineering, KGISL Institute of Technology, Coimbatore, Tamilnadu 641035, India; d Department of Mechanical Engineering, MarEphraem College of Engineering and Technology, Marthandam, Tamil Nadu 629171, India; e Department of Mechanical Engineering, Sri Krishna College of Technology, Coimbatore, Tamilnadu 641042, India

**Keywords:** Additive manufacturing, Natural fiber, Filament, 3D printing, Biodegradability, Fabrication and Testing methods of Sustainable Natural Fiber FIlaments

## Abstract

The rapid advancement of additive manufacturing (AM), or 3D printing, has created unparalleled prospects for design innovation and production efficiency. Nonetheless, dependence on petroleum-derived polymers presents considerable environmental and sustainability issues. In reaction to these issues, natural fiber-reinforced filament technologies have surfaced as a promising approach for sustainable production. This review aims to objectively evaluate the progress in the development of natural fiber-reinforced filaments, emphasizing their capacity to diminish the carbon footprint of additive manufacturing while improving mechanical, thermal, and functional characteristics. This paper systematically examines manufacturing methodologies, including pelletization and extrusion processes, material behavior such as viscoelasticity and morphological characteristics, and assesses their performance in various applications within the automotive, aerospace, medical, and consumer goods sectors. Particular attention is directed on resolving enduring challenges including material inconsistency, moisture sensitivity, and recycling constraints. Moreover, the analysis delineates existing research deficiencies and suggests prospective avenues, including the incorporation of smart materials and the implementation of circular economy frameworks. This article provides a thorough assessment of difficulties and opportunities to facilitate the progress and practical implementation of sustainable natural fiber-based filaments in the next generation of additive manufacturing.

## Review Highlights

• This review examines the revolutionary potential of natural fiber-reinforced filaments in enhancing sustainable FDM 3D printing.
• Examines current advancements in pelletized natural fiber composites, focusing on issues such as moisture control and mechanical properties.
• Demonstrates the adaptability of natural fiber filaments across sectors including automotive, aerospace, and consumer products.
• Identifies research deficiencies and suggests improvements in recyclability, material uniformity, and scalable production for more sustainable additive manufacturing.

## Graphical abstract

**Figure:**
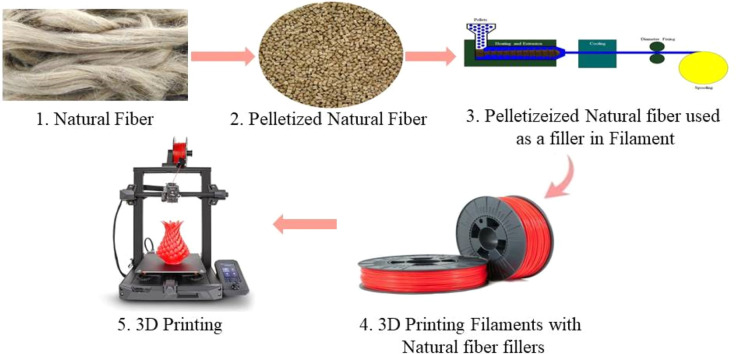

Specifications table

**Table:** 

Subject area:	Engineering
More specific subject area:	*Additive Manufacturing*
Name of the reviewed methodology:	*Fabrication and Testing methods of Sustainable Natural Fiber FIlaments Fabrication and Testing methods of Sustainable Natural Fiber FIlaments*
Keywords:	*Additive manufacturing, Natural fiber, Filament, 3D printing, Biodegradability*
Resource availability:	• Y. Pasha Shaik, J. Schuster, A. Shaik, A Scientific Review on Various Pellet Extruders Used in 3D Printing FDM Processes, J. 2021. 8 (2021) 7698. https://doi.org/10.4236/oalib.1107698. • T. Hachimi, N. Naboulsi, F. Majid, R. Rhanim, I. Mrani, H. Rhanim, Design and Manufacturing of a 3D printer filaments extruder, Procedia Struct. Integr. 33 (2021) 907-916. https://doi.org/10.1016/J.PROSTR.2021.10.101. • H. He, K. Molnár, Fabrication of 3D printed nanocomposites with electrospun nanofiber interleaves, Addit. Manuf. 46 (2021) 102030. https://doi.org/10.1016/J.ADDMA.2021.102030. • A.I. Hofmann, I.O.¨ Stergren, Y. Kim, S. Fauth, M. Craighero, M.-H. Yoon, A. Lund, C. Mü, All-Polymer Conducting Fibers and 3D Prints via Melt Processing and Templated Polymerization, Cite This ACS Appl. Mater. Interfaces. 12 (2020) 8721. https://doi.org/10.1021/acsami.9b20615. • L.B. Silva, R.O. de Oliveira, G.F. Barbosa, S.B. Shiki, K. Fu, Influence of the single-screw extruder nozzle diameter on pellet-based filaments for additive manufacturing, J. Brazilian Soc. Mech. Sci. Eng. 44 (2022) 1-11. https://doi.org/10.1007/s40430-022-03590-z. • S. Bandari, D. Nyavanandi, N. Dumpa, M.A. Repka, Coupling hot melt extrusion and fused deposition modeling: Critical properties for successful performance, Adv. Drug Deliv. Rev. 172 (2021) 52-63. https://doi.org/10.1016/J.ADDR.2021.02.006. • F. Zavřel, M. Novák, J. Kroupová, C. Beveridge, F. Štěpánek, G. Ruphuy, Development of Hot-Melt Extrusion Method to Produce Hydroxyapatite/Polycaprolactone Composite Filaments, Adv. Eng. Mater. 24 (2022) 2100820. https://doi.org/10.1002/ADEM.202100820. • H.Y. Lau, M.S. Hussin, S. Hamat, M.S. Abdul.Manan, M. Ibrahim, H. Zakaria, Effect of kenaf fiber loading on the tensile properties of 3D printing PLA filament, Mater. Today Proc. (2023). https://doi.org/10.1016/J.MATPR.2023.03.015. • D. Stoof, K. Pickering, Y. Zhang, Fused Deposition Modeling of Natural Fibre/Polylactic Acid Composites, J. Compos. Sci. 2017, Vol. 1, Page 8. 1 (2017) 8. https://doi.org/10.3390/JCS1010008.
Review question:	1. What are the key benefits of using natural fiber-reinforced filaments over traditional petroleum-based 3D printing materials? 2. How does the pelletization process improve the manufacturability and performance of natural fiber filaments for FDM 3D printing? 3. What are the current challenges in achieving consistent mechanical and thermal properties in natural fiber-reinforced filaments? 4. How can advancements in moisture management and recycling strategies enhance the applicability of natural fiber filaments in various industries? 5. What future research directions and innovations could address the scalability and industrial adoption of sustainable 3D printing materials?

## Background

Additive manufacturing, commonly referred to as three-dimensional (3D) printing, has become a disruptive technology with a broad range of applications across multiple industries [1,2]. In contrast to conventional subtractive manufacturing, which involves removing materials from a solid block, 3D printing creates objects layer by layer, providing previously unheard-of efficiency and design freedom [[3], [4], [5]]. The additive layering of material is the basic idea behind 3D printing. First, a digital three-dimensional model is produced, usually with the use of computer-aided design (CAD) software. This virtual model is divided into multiple horizontal cross-sections, acting as a construction blueprint for the real object [[6], [7], [8]]. The 3D printer, a device that converts digital designs into physical objects, is essential to 3D printing. These printers are available in a range of sizes and configurations, from hobbyist-friendly desktop models to large-scale industrial systems for intricate applications [9,10]. A vast variety of materials are used by 3D printers, such as composites, metals (like titanium and aluminum), plastics (like PLA and ABS), ceramics, and even biological materials (like living cells). The intended uses and qualities determine the material selection. Layer by layer, material is deposited or fused in accordance with the specifications of the digital model during the core process. Until the finished object is complete, this layering process is repeated [11,12].

Numerous industries and fields have seen radical changes thanks to 3D printing: it is now affordable for small-batch and one-off manufacturing due to its ability to produce complex, customised parts with minimal waste and to enable rapid prototyping [[13], [14], [15], [16]]. Fuel consumption and efficiency are improved when lightweight, high-performance parts for aircraft and spacecraft are produced using 3D printing. For example, NASA has successfully 3D printed rocket engine components such as the fuel injector and combustion chamber, while SpaceX has utilized 3D printing to manufacture the SuperDraco engine chambers for their Dragon spacecraft [17,18]. It makes the ability to create implants tailored to each patient in the medical field [[19], [20], [21]], prosthetics, and even functional organs through bioprinting [[22], [23], [24]]. Large-scale 3D printers can create building components, reducing construction time and material waste [25,26]. Designers and artists use 3D printing to create innovative prototypes, clothing, and elaborate sculptures. Students can visualize complex concepts and make STEM (science, technology, engineering, and mathematics) learning more engaging with the help of 3D printing, which is an invaluable teaching tool [27]. Even though 3D printing has a lot of potential, there are still issues to be resolved, such as scalability issues, material limitations, and the requirement for better quality control. However, on-going research and development hold out hope for resolving these problems and broadening the application of the technology [28,29].

In order to address environmental concerns, reduce waste, and promote eco-friendly manufacturing practices, the use of sustainable materials in 3D printing is extremely important. Using these materials in 3D printing processes has several important advantages as industries come to understand the value of sustainability [30,31]. Recycled filaments and bio plastics are examples of sustainable materials that lessen the carbon footprint of 3D printing. From production to disposal, they frequently have a smaller environmental effect throughout their lifecycle. By using sustainable materials, precious resources like metals and plastics derived from petroleum are preserved. Because they are frequently derived from recycled or renewable resources, fewer virgin resources are needed. Material waste from traditional subtractive manufacturing processes is substantial [32,33]. On the other hand, additive manufacturing in 3D printing using sustainable materials results in little to no waste. This makes the economy more resource-efficient and circular. Reducing the overall energy consumption of 3D printing processes can be achieved by using sustainable materials during production [34,35]. A smaller environmental impact and cheaper operating costs are two benefits of this energy efficiency. Recyclable or biodegradable, a lot of sustainable materials allay worries about plastic pollution. Filters that are biodegradable decompose organically, whereas materials that are recycled encourage closed-loop systems that minimize waste production. Local manufacturers are able to produce customized, on-demand goods thanks to sustainable materials [36,37]. As a result, there is less need for long-distance shipping and warehousing, which further lowers the transportation-related carbon emissions. Innovation in material science is fuelled by the creation and application of sustainable materials in 3D printing. In order to contribute to a more sustainable future, researchers are constantly investigating new environmentally friendly materials and manufacturing processes. Growing consumer awareness of environmental issues has led to a preference for products made of sustainable materials [38,39]. Using these materials for 3D printing is in line with consumer preferences and could give companies a competitive edge. Stricter sustainability and environmental standards are being implemented by governments and regulatory agencies. Businesses are better positioned to comply with these regulations and stay out of trouble by using sustainable materials for 3D printing. Businesses that integrate sustainability into their manufacturing processes frequently see an improvement in their brand recognition and client retention. Using sustainable materials in 3D printing can improve a business's standing as an environmentally responsible organization [40,41].

Natural fiber reinforced filaments, which provide sustainable substitutes for conventional plastic-based materials, represent a revolutionary approach to 3D printing. An overview of natural fiber reinforced filaments, including their composition, benefits, and importance in the field of additive manufacturing, is given in this introduction [[42], [43], [44]]. Composite materials made of natural fiber reinforced filaments primarily consist of two parts: Organic Fibers: Plant-based, renewable resources like bamboo, hemp, flax, sisal, jute, cotton, and agricultural waste materials like rice husks and sugarcane bagasse are the source of these fibers. Because of their high strength-to-weight ratio, low environmental impact, and biodegradability, natural fibers are preferred [[45], [46], [47]]. Polymer Matrices: Polymer matrices, usually biodegradable thermoplastics or polylactic acid (PLA), are mixed with natural fibers. The polymer acts as a binding agent, enhancing the mechanical properties and printability of the composite material [[48], [49], [50]].

This review paper aims to deliver a thorough overview of improvements in natural fiber reinforced filament technology for 3D printing. It seeks to emphasize the viability of natural fibers as sustainable substitutes for traditional petroleum-derived materials, concentrating on their mechanical, thermal, and ecological characteristics. The paper examines the fabrication techniques, such as pelletization and extrusion processes, that facilitate the incorporation of natural fibers into polymer matrices for additive manufacturing, Table 1. This work's novelty is in its comprehensive approach to tackling challenges including material uniformity, moisture control, and recyclability, while investigating innovative solutions and future trends. This essay establishes a basis for more sustainable and efficient additive manufacturing techniques by incorporating concepts from other disciplines, highlighting natural fiber filaments as revolutionary materials in 3D printing.

**Table 1:** Currents issues in FDM Additive Manufacturing and Advancements in current work.

Aspect	Current Issue	Research Gap	Advancements in Current Work
Material Properties	Limited mechanical strength and thermal stability of traditional FDM materials.	Inconsistent reinforcement of natural fibers in filaments; lack of understanding of optimal fiber-polymer interaction.	Development of pelletized natural fiber filaments with tailored fiber distribution to improve strength and stability.
Sustainability	High reliance on petroleum-based polymers contributing to environmental degradation.	Limited adoption of biodegradable and recyclable materials in FDM applications.	Introduction of biodegradable polymer-natural fiber composites to reduce carbon footprint in additive manufacturing.
Moisture Management	Natural fibers absorb moisture, leading to filament degradation and printing defects.	Lack of effective strategies for moisture control during filament production and storage.	Pelletization techniques to reduce moisture content and improve material performance during extrusion.
Printability	Natural fiber composites face issues like nozzle clogging and uneven extrusion during printing.	Insufficient research on optimizing extrusion parameters for fiber-reinforced filaments.	Advanced extrusion methods ensuring consistent filament flow and enhanced printability for complex geometries.
Material Recycling	Limited recyclability of conventional filaments; disposal of unused raw materials poses environmental risks.	Lack of effective recycling strategies for natural fiber reinforced composites.	Integration of circular economy principles with improved recyclability of fiber-reinforced FDM filaments.
Cost Efficiency	High production costs of alternative sustainable filaments compared to traditional options.	Limited scalability of sustainable filament production for industrial applications.	Economical fabrication processes, such as pelletization, making natural fiber filaments viable for large-scale production.
Testing and Validation	Inadequate evaluation of long-term performance and durability of natural fiber reinforced filaments.	Limited standardized testing protocols for assessing mechanical and environmental properties of these materials.	Comprehensive testing on mechanical, thermal, and morphological properties to validate filament performance.
Application Potential	Limited adoption of natural fiber filaments in high-performance industries like automotive and aerospace.	Lack of data on industry-specific performance benchmarks for natural fiber composites.	Demonstration of use cases in automotive, consumer goods, and medical applications, showcasing material versatility.

### Natural fiber reinforced filaments

Rather than being created artificially, natural fibers are materials that are harvested or extracted from plants, animals, or geological processes. Humans have been using these fibers for thousands of years for a variety of uses, such as building, textiles, clothes, and more [[51], [52], [53]]. Many natural fibers come from plants, such as cotton (from cotton plants), jute (from the jute plant), flax (from flax plants), and hemp (from the hemp plant) [54,55]. Some natural fibers are derived from animals, like wool (from sheep), silk (from silkworms), and cashmere (from goats) [[56], [57], [58]]. Asbestos is a natural mineral fiber that has been used in construction and insulation, although it is now banned in many countries due to health concerns [59]. Since natural fibers can be restored by animal husbandry or with each plant growth season, they are usually considered renewable resources. Compared to synthetic fibers derived from petroleum-based materials, natural fibers are considered more environmentally friendly due to their faster biodegradation rates. For example, flax fibers have been shown to degrade within a few months under composting conditions, while synthetic fibers like polypropylene can take several decades to break down. When compared to synthetic fibers, they contribute less to environmental pollution because they are biodegradable, which means they break down naturally over time. Certain natural fibers, such as hemp and jute, are valued for their robustness and longevity, which makes them appropriate for a range of uses, such as building materials and textiles [60,61]. Natural fibers are commended for being breathable and comfortable. Natural fiber fabrics, like cotton and linen, allow air to flow through them, which makes them perfect for clothing in warm, muggy weather. Since many natural fibers are good at wicking away moisture, they are a good choice for towels, bedding, and sportswear. Natural fibers derived from animals, such as wool, act as great insulators and keep people warm in the winter. Wool has the ability to control body temperature and draw moisture away from the skin. The variety of textures and looks that natural fibers can provide enhances the visual appeal of textiles and other products. For instance, silk is renowned for having a posh feel and sheen. They are adaptable and can be applied to a variety of materials, including composite materials, paper, textiles, rope, and cordage [62,63]. Certain natural fibers can become more environmentally friendly by using less chemicals and pesticides when farming is done organically. Natural fiber prices and availability can change based on demand, climate, and geographic location, among other things. For instance, cotton is generally accessible and reasonably priced, but due to limited supply, fibers like cashmere may cost more. In numerous cultures and historical eras, natural fibers have had important roles. Silk, for instance, has long been associated with trade and luxury. The textile and materials industry still depends heavily on natural fibers, and ongoing research looks for ways to produce them in a way that is even more environmentally friendly and sustainable [64,65].

### Types of natural fibers used in filaments

**Fig. 1:**
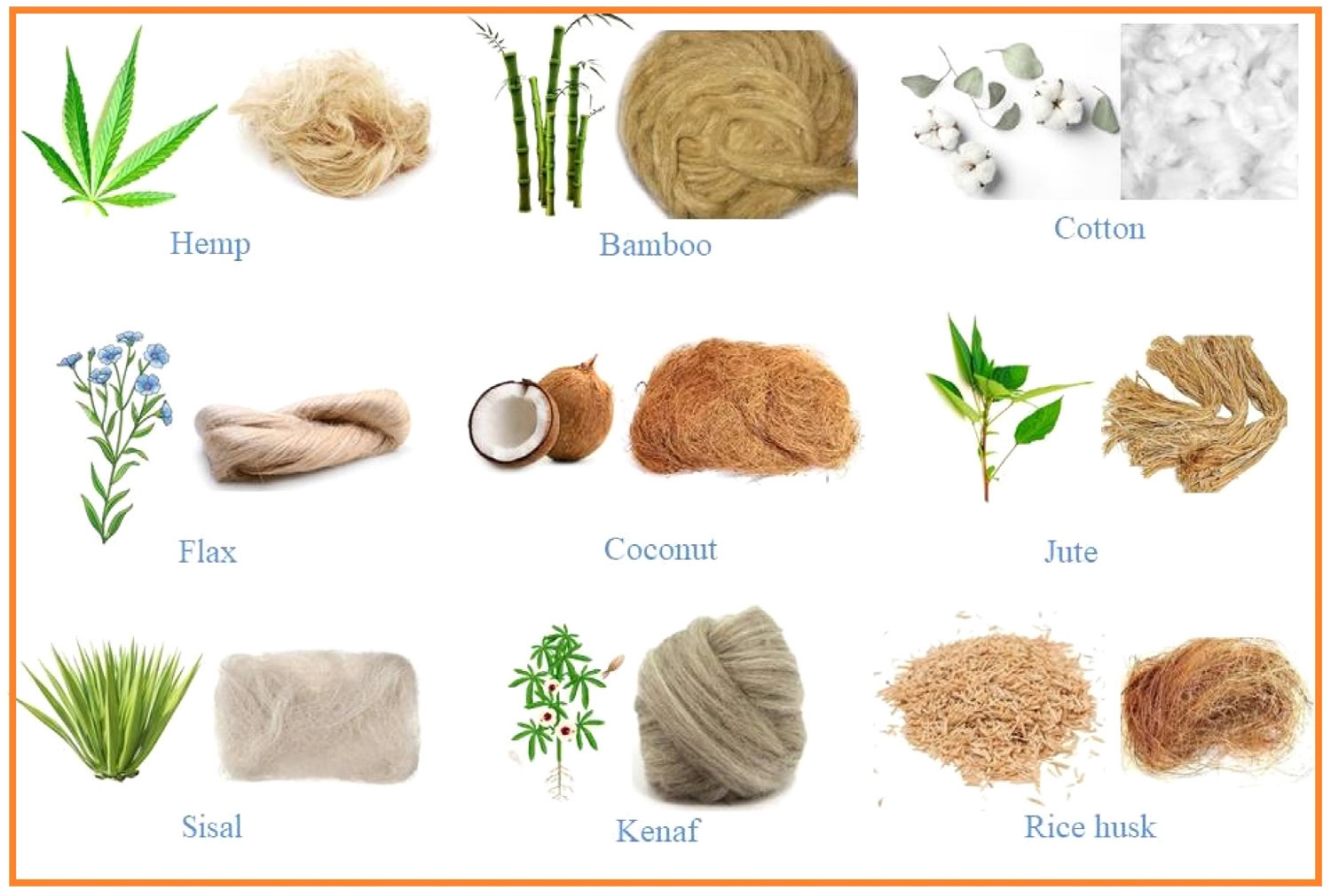
Types of Natural fiber that can be used in 3D printing filament.

Natural fibers provide a more environmentally friendly option to traditional plastics when used in 3D printing filaments. By combining these fibers with polymer matrices, composite filaments with increased strength and sustainability are produced. Fig. 1 shows the different types of natural fiber that can be used in 3D printing filament.

#### Hemp fiber

The hemp plant, renowned for its quick growth and little effect on the environment, is the source of hemp fibers. Filaments made of hemp are biodegradable, lightweight, and have good strength qualities. They can be used for a variety of purposes, such as eco-friendly products, artistic creations, and prototypes [66,67].

#### Flax fiber

The flax plant yields biodegradable and renewable flax fibers. Filaments made from flax have a distinctive look and a naturally textured surface. Prototypes as well as decorative and artistic prints are made with them [68,69].

#### Jute fiber

The jute plant, which is prized for its affordability and sustainability, yields jute fibers. Filaments made of jute are biodegradable and have a high tensile strength. They are used in packaging, home décor, and environmentally friendly goods [70,71].

#### Bamboo fiber

Bamboo plants, which grow quickly and are renewable, are the source of bamboo fibers. Filaments made of bamboo have a distinctive look that resembles wood. They are utilized in creative endeavors, ornamental prints, and environmentally conscious designs [72,73].

#### Cotton fiber

Even though cotton fibers are frequently used in textiles, they can also be combined with PLA to produce filaments of natural fiber. Filaments made of cotton provide a smooth, textile-like surface, making them ideal for 3D prints that resemble fabric [74].

#### Kenaf fiber

The kenaf plant, which grows quickly and sustainably, is the source of kenaf fibers. Filaments made of kenaf are biodegradable and have good strength characteristics. They are utilized in many different contexts, such as packaging and environmentally friendly goods [75,76].

#### Coconut fiber

The husk of coconuts is where coconut fibers, also known as coir fibers, originate. Filaments made of coir are strong, biodegradable, and lightweight. They are used in creative prints and goods that need to have a natural aesthetic [77,78].

#### Sisal fiber

The sisal plant, which is prized for its sturdiness and strength, yields sisal fibers. Filaments made of sisal have good mechanical qualities and are biodegradable. They are utilized in environmentally conscious designs and useful prototypes [79].

#### Abaca fiber

Abaca fibers resemble banana fibers and are derived from the abaca plant. Filaments made of abaca are flexible and strong, and they biodegrade. They are employed in many different contexts, such as creative prints [80].

#### Algae fiber

Algal biomass is the source of these relatively new algae-based filaments. - Algae filaments have the potential to enable sustainable 3D printing because they are biodegradable. They are employed in studies and experiments looking into new sustainable materials [81,82].

These natural fibers provide biodegradable substitutes for conventional plastics, which makes them an eco-friendly and sustainable addition to filaments for 3D printing. Additionally, their distinctive textures and beauty open up possibilities for imaginative and artistic applications in additive manufacturing. Table 2 indicates the mechanical properties of different natural fibers.

**Table 2:** Mechanical properties of Natural Fibers.

Fibers	Density (g/cm^3^)	Tensile Strength (MPa)	Young’s Modulus (GPa)	Elongation %
Hemp Fiber	1.4	443	23	1.6
Flax Fiber	1.52	613	59	2.4
Jute Fiber	1.5	500	20	1.51
Bamboo Fiber	0.6	585	15.7	6.3
Cotton Fiber	1.55	410	8	6
Kenaf Fiber	1.52	780	22	12.5
Coconut Fiber	1.3	350	5	40
Sisal Fiber	1.45	355	11.04	3.02
Abaca Fiber	1.5	750	41	7
Algae Fiber	1.06	215	24	4

### Advantages of natural fiber reinforced filaments

Natural fiber reinforced filaments are a desirable option for many applications because they provide a number of benefits in 3D printing. These benefits result from the integration of polymer matrices with natural fibres. Natural fibers are more environmentally friendly and sustainable than materials based on petroleum because they come from renewable sources like plants [83,84]. These filaments contain a large number of compostable or biodegradable natural fibers and biodegradable polymers, which lessens their impact on the environment and plastic waste. When compared to synthetic materials, the production of natural fibers usually has a lower carbon footprint, which helps mitigate climate change. Natural fiber filaments help lower greenhouse gas emissions by minimizing the use of fossil fuels in manufacturing. Tensile strength and impact resistance are two examples of the mechanical qualities of the resultant filament that can be markedly enhanced by integrating natural fibers into polymer matrices. Natural fiber reinforced filaments are lightweight despite having increased strength, which makes them appropriate for uses where weight is an issue, like in the aerospace industry [85,86]. A variety of natural fibers, each with their own special qualities, such as hemp, flax, jute, cotton, and more, can be used to create natural fiber reinforced filaments. These filaments can be used for a wide range of products, including industrial and construction components, consumer goods, and automotive parts. Natural fiber reinforced filaments are ideal for artistic and decorative applications because of their distinctive, natural look, which enhances the visual appeal of printed objects. Natural fibers can prolong the life of 3D printer components because they are typically less abrasive than some conventional composite reinforcements (like carbon fibers). Compared to certain specialty filaments, natural fibers are frequently more affordable and easily obtainable, meaning that material costs are decreased. When processed correctly, natural fiber reinforced filaments show less warping during printing than some conventional filaments, such as ABS. Because of their many uses, these filaments can be found in a wide range of industries, including consumer goods, automotive, aerospace, construction, and even medical devices. In the field of 3D printing, natural fiber reinforced filaments are becoming more and more popular as an eco-friendly and effective replacement for conventional materials. They provide improved material properties for a variety of applications and are in line with the increasing demand for creative and environmentally friendly solutions [[87], [88], [89]].

## Method details

### Pelletization process of natural fibers

**Fig. 2:**
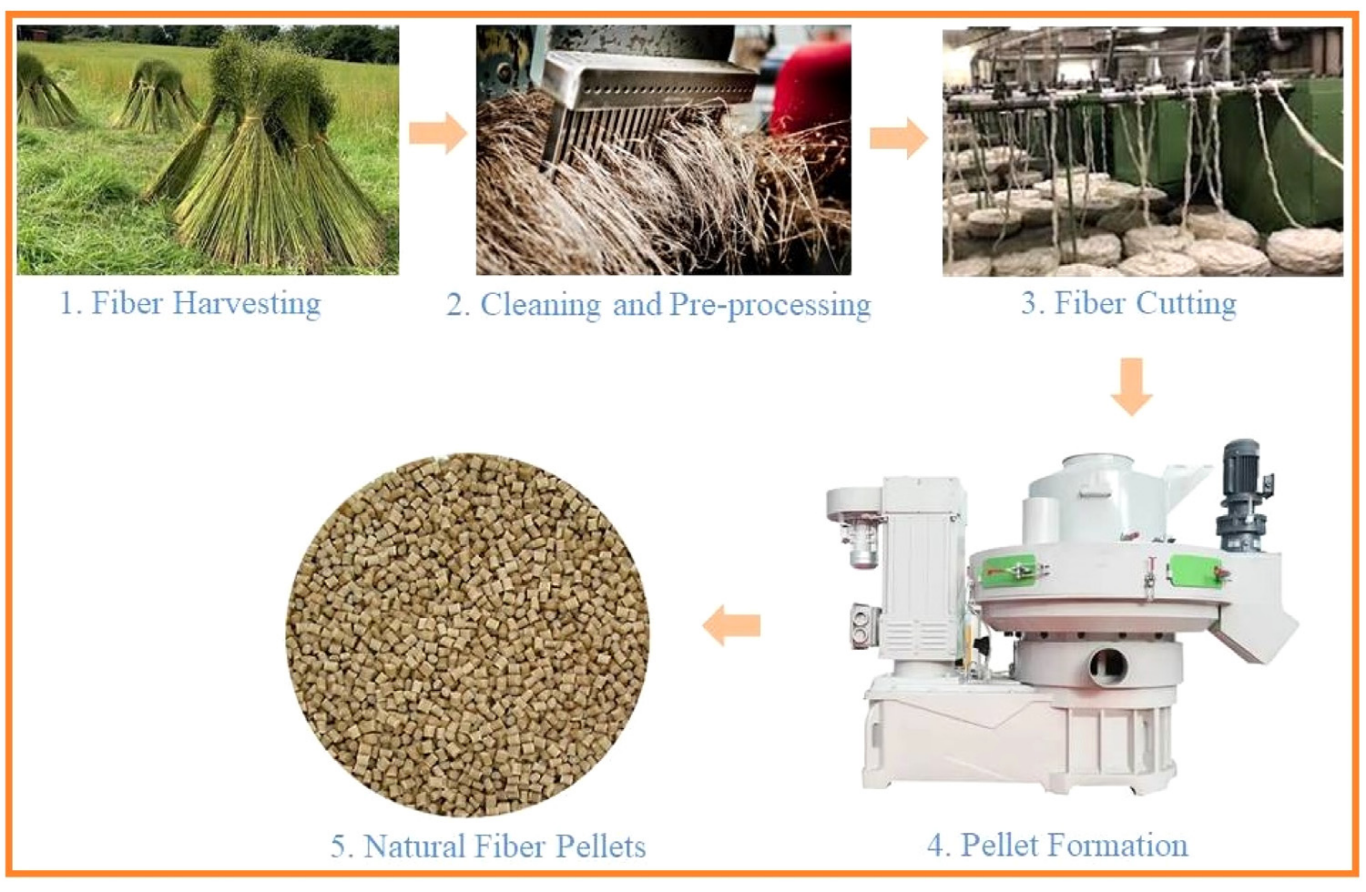
Pelletization process of Natural Fiber.

The pelletization process for natural fibers involves converting raw natural fibers into small, uniform pellets or granules that are suitable for use as feedstock in 3D printing filament production (Fig. 2). This process offers several benefits, making it a crucial step in the utilization of natural fibers in additive manufacturing [[90], [91], [92]].

The initial phase of preparation entails procuring raw natural fibers from botanical or zoological sources, like flax, hemp, bamboo, or wool. These fibers frequently contain dust, waxes, and other contaminants, requiring a pre-processing stage that involves washing, drying, and the elimination of non-fibrous materials. To maintain uniformity and facilitate processing, the fibers are then truncated into shorter segments. The regulation of fiber length is generally accomplished using precision cutting or milling machinery, with the desired length typically falling between 1-3 mm, which is optimal for uniform distribution in polymer matrices and suitable for pelletizing systems. The short fibers are subsequently introduced into a pelletizer, where they are combined with a thermoplastic polymer, and the amalgamation is subjected to heating, compression, and extrusion into cylindrical pellets. These pellets are typically chilled and chopped to standard dimensions of 2-5 mm in diameter, which are ideal for material extrusion (MEX) 3D printing machines and compatible with the majority of single- and twin-screw extruders. Research is progressing towards the development of continuous natural fiber filaments, in addition to the preparation of short fiber pellets. Methods including fiber coating, pultrusion techniques, and direct thermoplastic impregnation are being investigated to produce filaments reinforced with continuous flax, jute, or hemp fibers, with the objective of improving mechanical performance in printed components via enhanced load transfer and fiber alignment [[93], [94], [95]].

### Benefits of pelletization: transforming natural fibers into usable form

A crucial step in using natural fibers for a variety of purposes, including 3D printing, is pelletization. With this method, naturally occurring loose and irregular fibers are transformed into a more consistent and manageable form, usually in the form of tiny pellets or granules. Natural fibers are easier to handle and feed into industrial machinery, such as 3D printers, when they are converted into pellets. This streamlines the production procedure. Because of their uniform shape and compact size, pellets store well. They are more economical to store and transport because they take up less room than loose fibers. Transporting pellets is more practical. They can be packaged effectively, which lowers the volume and weight of materials in transit and helps to save money on transportation. Pelletization standardizes the properties of natural fibers, such as size, moisture content, and impurities. This consistency ensures a higher quality feedstock for 3D printing and other applications [96,97]. The processing and properties of natural fibers can be impacted by their susceptibility to moisture absorption. Pelletization improves the stability and printability of the materials by lowering their moisture content. Fibers can be cut to a consistent length during the pelletization process, giving manufacturers control over the length distribution. The final product's printability and mechanical qualities are impacted by this exactness in fiber length. Natural fibers and polymer matrices interact better when pelletization is used. Better bonding is the outcome, which improves the mechanical qualities and general product quality. Improved flow properties in pelletized fibers allow for smoother and more reliable filament extrusion during the extrusion process. This lessens the possibility of printing flaws and nozzle clogs in 3D printing. Natural fibers can be produced on a large scale in pelletized form, which enables them to be used for industrial applications in the construction, automotive, and aerospace industries. Pelletization converts loose fibers into pellets that can be used, minimizing material waste. As a result, less wasted or extra raw fiber is disposed of, supporting sustainability objectives [[98], [99], [100]].

**Fig. 3:**
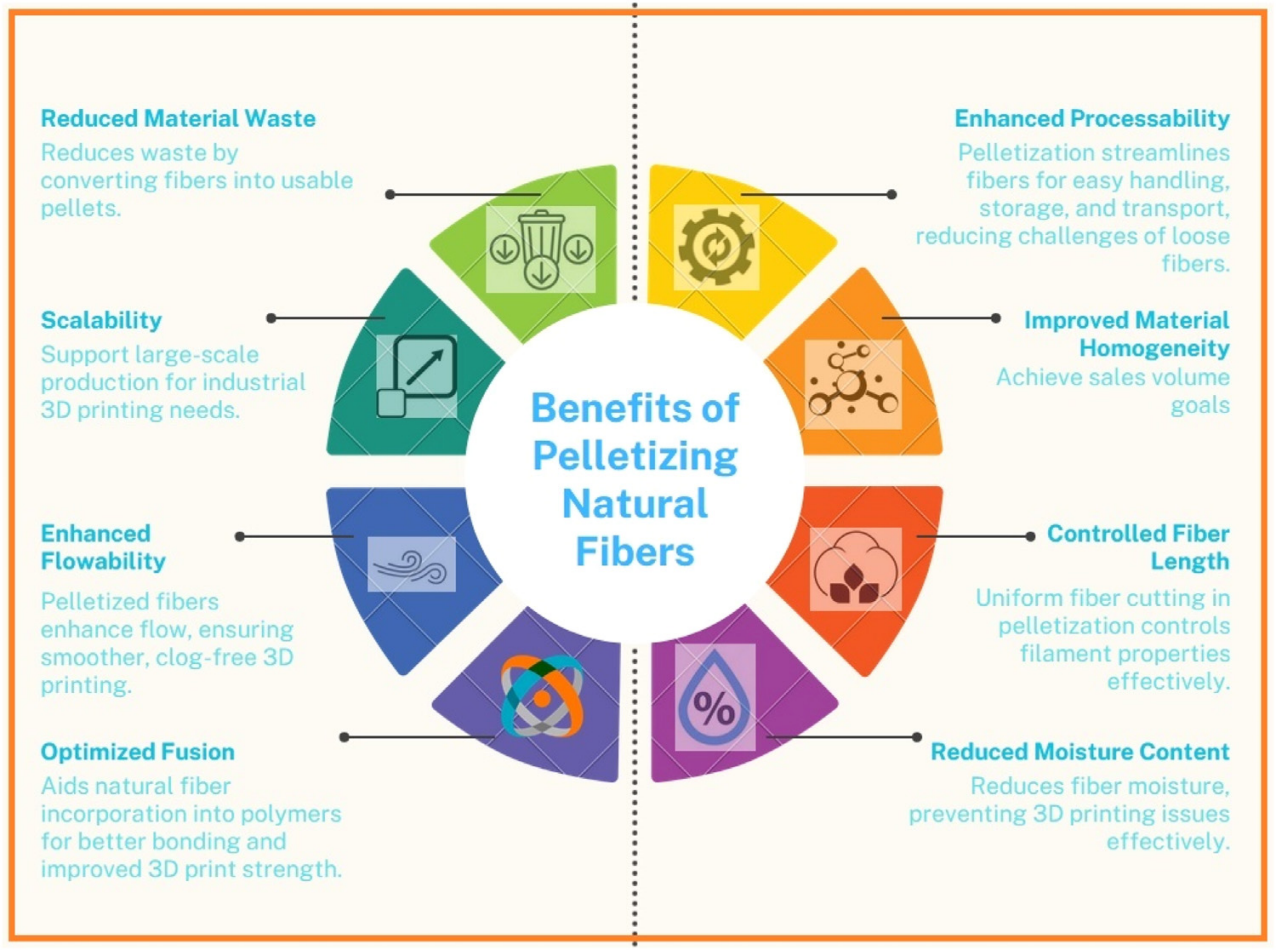
Benefits of pelletizing natural fiber.

Pelletization plays a vital role in unlocking the potential of natural fibers, offering numerous advantages that enhance their usability in a wide range of applications (Fig. 3). This process not only improves the material's handling, storage, and processing but also contributes to more sustainable and efficient manufacturing practices.

## Manufacturing methods of pellets to 3D printing filaments

To transform natural fiber pellets into continuous fiber filaments for 3D printing, specific processing techniques are employed that allow the integration of long, uninterrupted fibers into a thermoplastic matrix. Initially, continuous natural fibers such as flax, jute, or hemp are selected in the form of rovings or yarns and treated using alkali, silane, or plasma methods to enhance fiber-matrix adhesion. Instead of relying on pelletized short fibers, these treated continuous fibers are fed into a modified extrusion system where they are co-extruded with molten biodegradable polymers like PLA in a core-sheath arrangement—forming a filament with the natural fiber as the core and the thermoplastic as the sheath [101,102]. This co-extrusion process ensures proper impregnation and alignment of fibers, significantly improving the mechanical strength, load-bearing capacity, and durability of the resulting 3D printed parts. Unlike short fibers, which are limited by discontinuous load transfer, continuous fibers enable uninterrupted stress distribution throughout the printed structure, making them suitable for structural and functional applications [103,104]. While such techniques require specialized equipment and careful control of thermal parameters to avoid fiber degradation, they offer a promising pathway for producing high-performance, eco-friendly composite filaments for advanced additive manufacturing [105].

### Fabrication techniques

The techniques employed to convert pellets into 3D printable filaments involve a series of steps to ensure uniformity, compatibility, and quality.

#### Extrusion

**Fig. 4:**
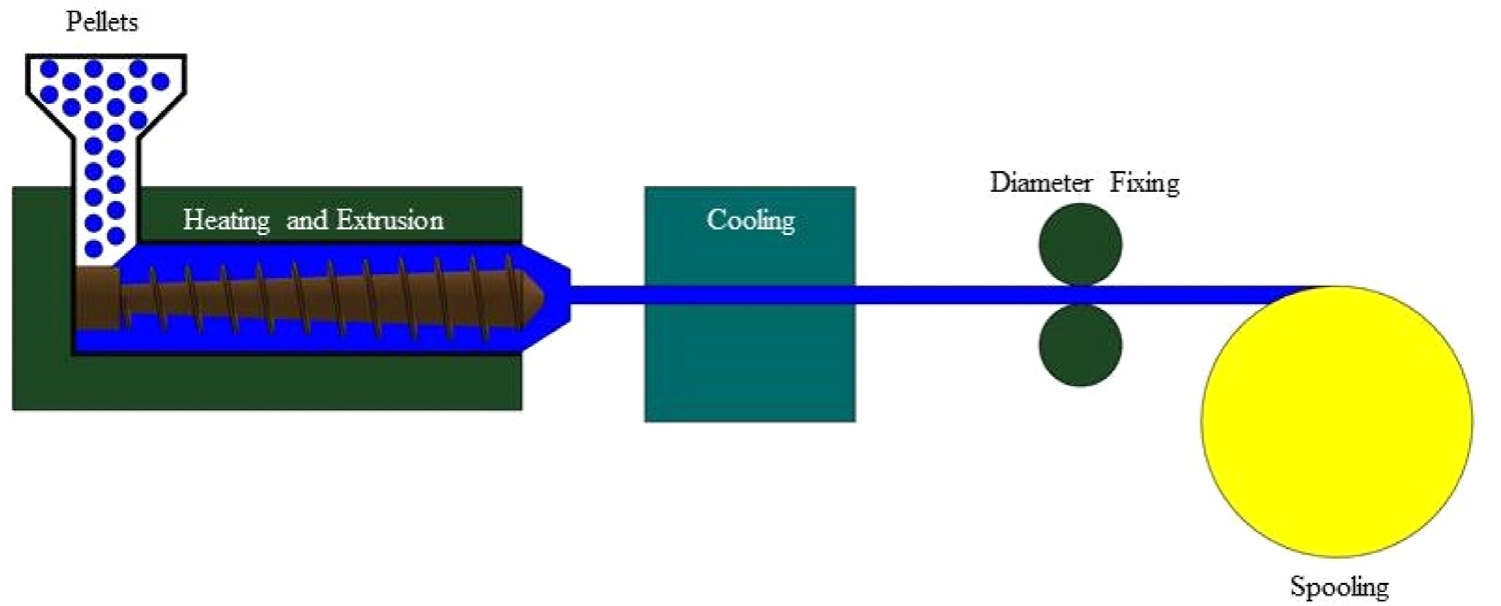
Extrusion method of fabricating filaments from pellets.

To make a continuous filament, pellets are heated to their melting point and then extruded through a die (Fig. 4). Diameter and consistency can be precisely controlled with this process. To solidify its size and shape, the extruded filament is quickly cooled, usually using water or air cooling. Sizing guarantees that the filament satisfies the required diameter requirements [106].

#### Melt spinning

**Fig. 5:**
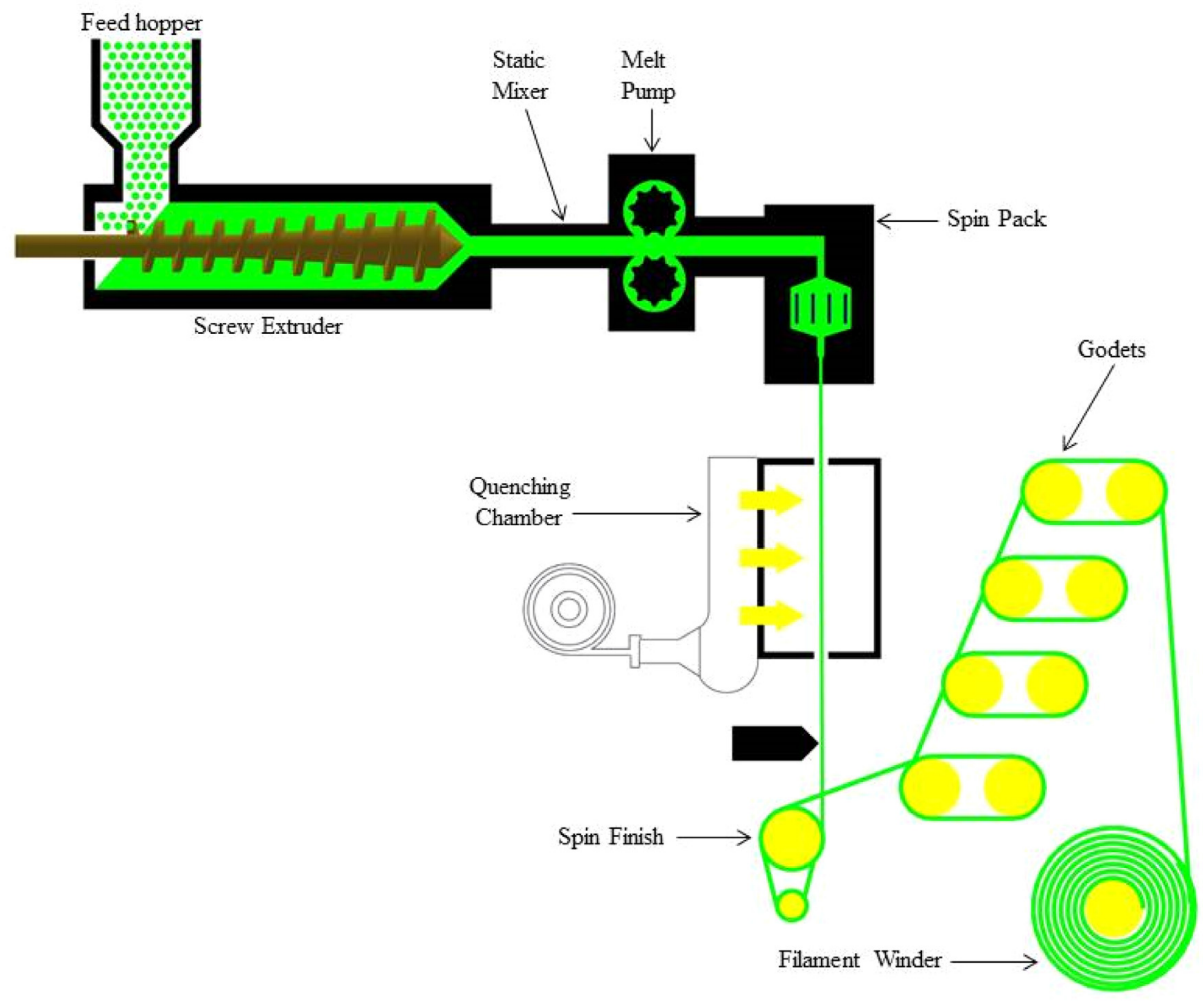
Melt spinning method of fabricating filaments from pellets.

The pellets are melted and then extruded through a spinneret or nozzle to form fine filaments (Fig. 5). Although this method is frequently applied to polymers, it can also be modified for materials based on natural fibres. After being quickly cooled and solidified, the extruded material forms continuous filaments that can be wound onto spools [107,108].

#### Twin-Screw extrusion

**Fig. 6:**
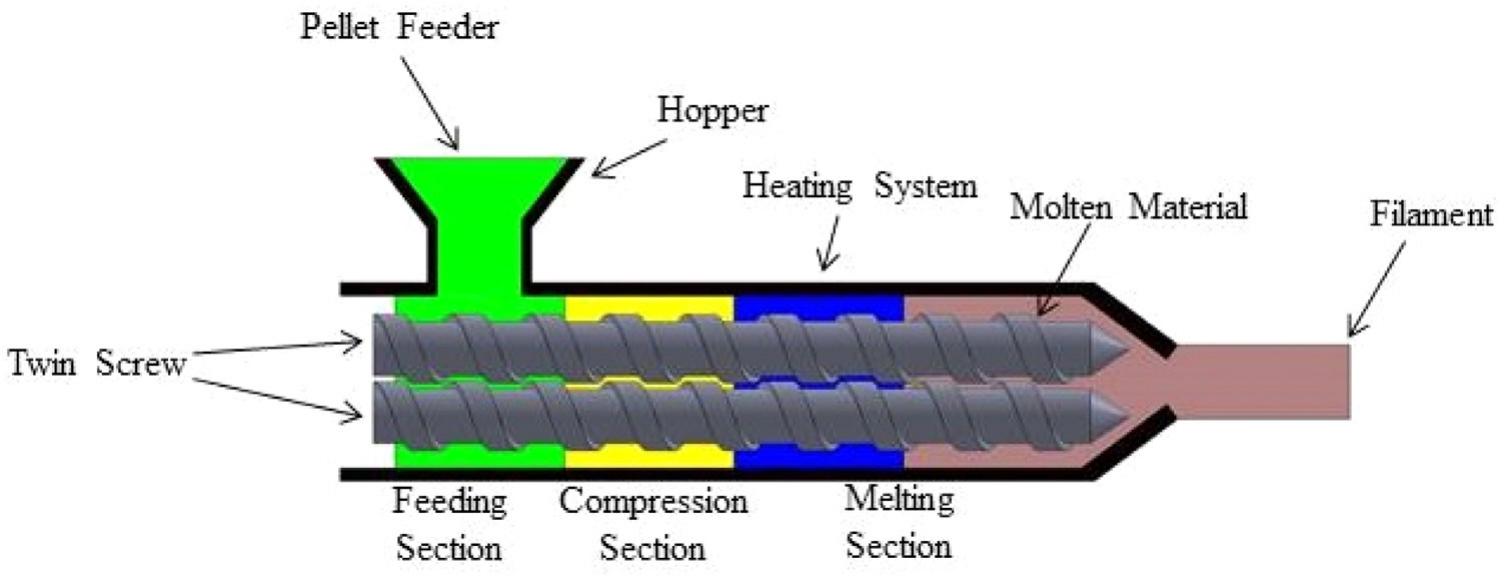
Twin-Screw Extrusion method of fabricating filaments from pellets.

The pellets with a polymer matrix are continuously mixed and melted using twin-screw extruders (Fig. 6). Comprehensive mixing and dispersing are guaranteed by the intermeshing screws. To create the filament, the combined material is extruded through a die, then cooled and sized as needed [109]. Twin-screw extrusion generates natural fiber-reinforced 3D printing filaments better than single-screw extrusion. Superior mixing and dispersion ensures homogeneous fiber distribution in the polymer matrix, which is essential for mechanical performance. In addition to improving fiber wetting and interfacial bonding, regulated temperature and shear reduce fiber deterioration. Twin-screw systems allow precise fiber, additive, and coupling agent dosing, increasing material formulation versatility. Twin-screw extrusion produces high-quality composite filaments with customized qualities for material extrusion (MEX) 3D printing due to these attributes.

#### Hot Melt Extrusion (HME)

**Fig. 7:**
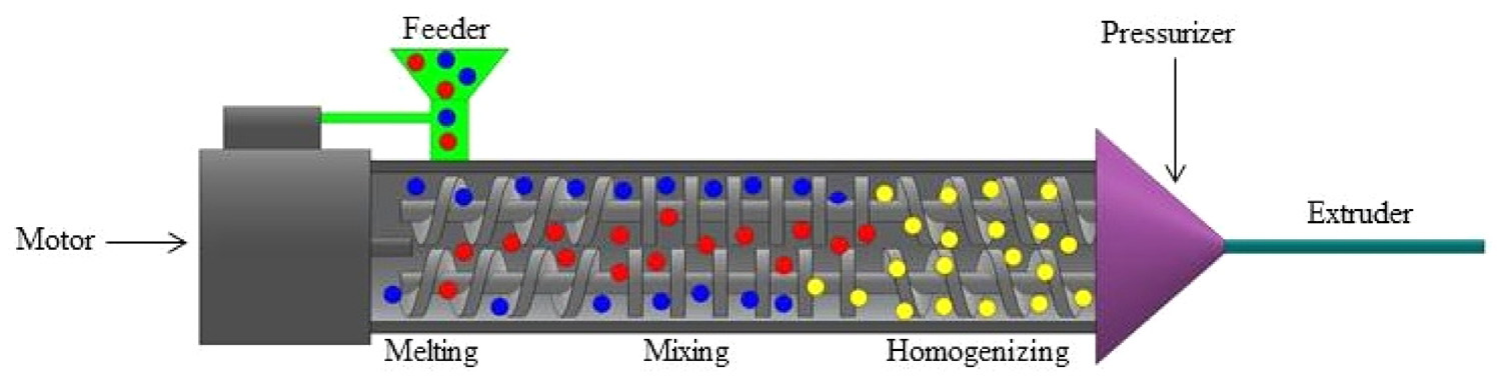
Hot melt Extrusion method of fabricating filaments from pellets.

Pellets soften and mix with polymers when exposed to a regulated temperature and pressure environment (Fig. 7). After that, the mixture is extruded to create filaments. After being extruded, the filament is quickly cooled and sized to the required diameter [110,111].

A filament manufacturing method's superiority depends on the natural fiber, polymer matrix, and filament application. Twin-screw extrusion is best for natural fiber-reinforced composites because to its mixing, fiber dispersion, and process control. Short fibers and thermoplastic matrices benefit from this approach because it minimizes fiber breakdown and ensures homogeneity. Single-screw extrusion, which is cheaper and easier to use, may be better for basic applications, but it cannot mix complex biocomposites. In-situ impregnation or co-extrusion are needed for continuous fiber reinforcement. Flax vs. bamboo and PLA vs. PP have a big impact on the process. Fibers with low thermal stability or hydrophilia will deteriorate or generate voids if not correctly handled, requiring softer, more controlled extrusion. To produce high-quality filaments, the manufacturing method must match the composite constituents' thermal, mechanical, and rheological properties. The selection of a technique is contingent upon various factors, including the intended filament properties, material composition, and production scale. Each technique possesses distinct advantages. To achieve the necessary strength, compatibility, and quality of the natural fiber-based filaments for 3D printing applications, a method's choice is essential.

## Material properties of natural fiber reinforced filaments

**Fig. 8:**
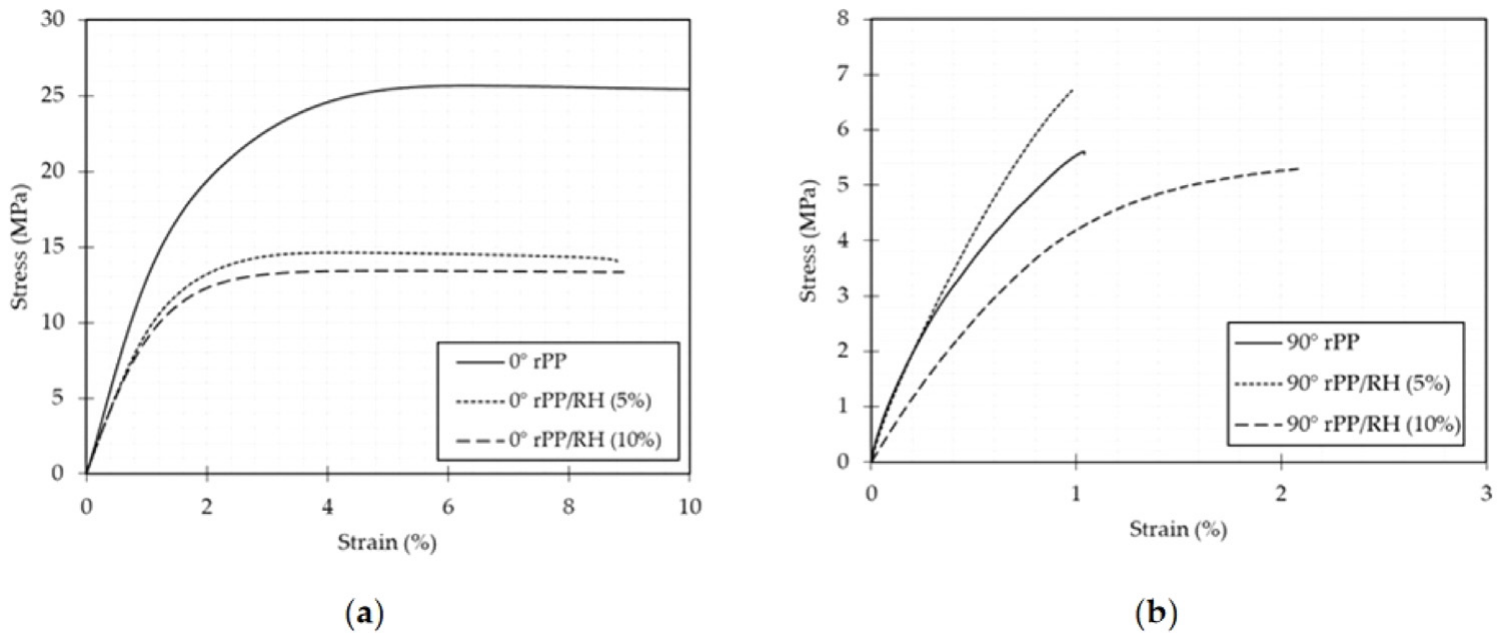
Stress–strain curves of 3D printed tensile specimens fabricated from polypropylene and rice husk polypropylene composites [114].

The mechanical properties of natural fiber reinforced filaments, which are used in 3D printing, are special and fit for a range of uses. The combination of natural fibers and polymer matrices produces these qualities. Filaments reinforced with natural fibers frequently have exceptional tensile strength, meaning they can bear pulling forces without breaking. The natural fiber type, alignment, and polymer matrix all affect the specific strength [112,113]. The mechanical behavior of 3D printed tensile specimens made of polypropylene and rice husk polypropylene composites is represented by stress-strain curves in Fig. 8. These specimens included either 5% or 10% weight of fiber and were oriented at 0° and 90°. The curves displayed distinct characteristics, starting from a linear elastic response and then moving into plastic deformation until the point of ultimate failure. Notably, consistent mechanical behaviors were revealed when the stress-strain curves for rice husk polypropylene composites were compared across various fiber weight ratios. This indicated an advantageous uniformity in fiber integration and potential variability management. Lightweight components are preferred for certain applications in the field of 3D printing filaments. The strain at which failure occurred in this situation was not significantly affected by the addition of 10% weight percent fiber. This finding implies that prototypes and filaments made with 10 weight percent fiber can be developed wisely without sacrificing their mechanical integrity. Table 3 explains the mechanical properties of natural fiber reinforced polymer filaments.

**Table 3:** Mechanical Properties of Natural fiber reinforced polymer filaments.

Natural Fiber	3D Printing Polymer Matrix	Tensile Strength (MPa)	Flexural Strength (MPa)	Young’s Modulus (GPa)	Key Observations
**Flax**	PLA	55–70	85–95	6.5–7.2	Good adhesion and printability; moderate stiffness.
**Hemp**	PLA	50–65	70–85	5.5–6.8	Biodegradable and printable; improved impact resistance.
**Jute**	PP	40–60	60–75	4.2–5.8	Cost-effective; moderate mechanical performance.
**Bamboo**	PLA	45–60	65–80	5.0–6.0	Good toughness and surface finish in prints.
**Basalt**	rPP	65–85	90–100	7.0–8.2	Superior strength; excellent interfacial bonding.

Improving the tensile strength of natural fiber-reinforced filaments and 3D-printed parts largely depends on enhancing the interfacial adhesion between the hydrophilic natural fibers and the typically hydrophobic polymer matrix. This can be achieved through various surface treatment methods that modify the fiber surface, thereby improving compatibility and load transfer. Chemical treatments such as alkali (NaOH) treatment remove lignin, hemicellulose, and surface impurities, exposing more cellulose and increasing surface roughness, which promotes mechanical interlocking with the polymer. Silane coupling agents are also widely used to form covalent bonds between fiber hydroxyl groups and the polymer, enhancing adhesion. Acetylation and maleic anhydride grafting (e.g., MAPP with polypropylene or MAPE with polyethylene) improve interfacial bonding by reducing fiber hydrophilicity. Plasma and enzymatic treatments offer environmentally friendly alternatives by modifying fiber surfaces without significant chemical use. These methods not only improve fiber-matrix adhesion but also contribute to better tensile properties and dimensional stability of the printed parts. Relevant studies such as those by Yan et al. (2022, Composites Part A) and Faruk et al. (2012, Progress in Polymer Science) support these techniques, reporting significant enhancements in mechanical performance through optimized surface modifications of natural fibers.

**Fig. 9:**
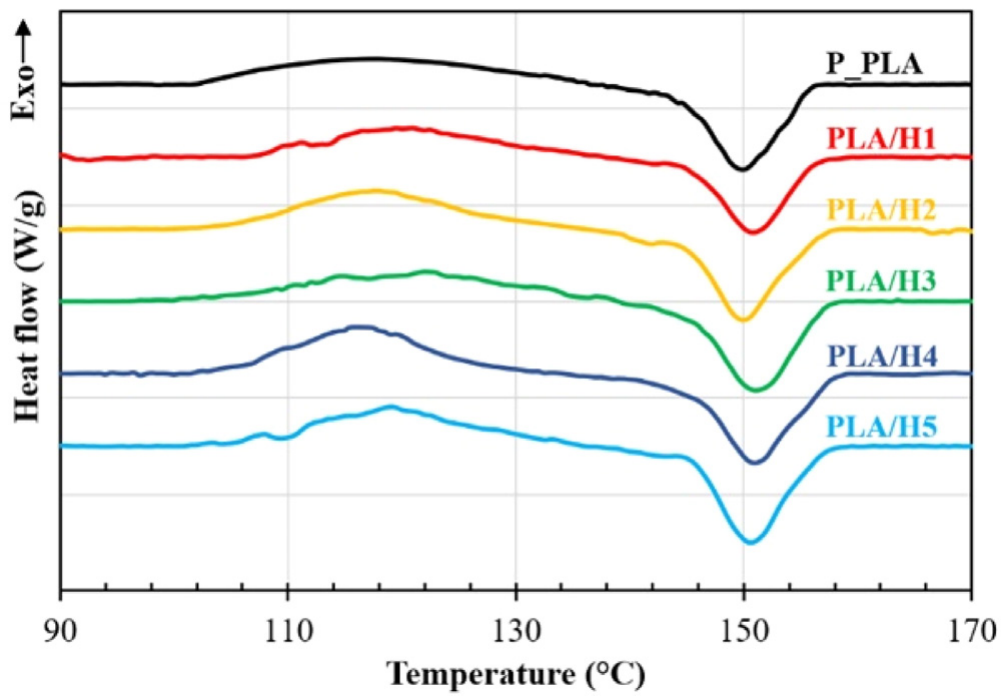
DSC heating curves of the printed PLA and henequen fibers reinforced PLA composite specimens [126].

The capacity of a material to bear bending without experiencing irreversible deformation or fracture is measured by its flexural strength. Natural fiber reinforced filaments are appropriate for applications needing flexibility and resilience because they can display good flexural strength [115]. These filaments can offer impressive impact resistance, making them ideal for parts and objects that may undergo sudden or repetitive loading without failure [116]. Compression strength refers to a material's ability to withstand axial loads (pushing or squeezing forces). Natural fiber reinforced filaments can exhibit reasonable compression strength, depending on the fiber type and composite composition [117,118]. Young's Modulus, a measure of a material's stiffness, indicates how resistant it is to deformation when a load is applied. While natural fiber reinforced filaments offer a balance between stiffness and flexibility, they frequently have a lower Young's modulus than certain synthetic composites. The capacity of a material to sustain repeated cyclic loading without failing is known as fatigue resistance. Natural fiber reinforced filaments are appropriate for applications involving dynamic stresses because they can provide good fatigue resistance [119,120]. The resistance of a material to bending, scratching, or indentation is measured by its hardness. Depending on their composition, natural fiber reinforced filaments can display a range of hardness levels [121,122]. Fracture toughness quantifies a material's resistance to crack propagation. Natural fiber reinforced filaments may have fracture toughness values that make them resilient to crack initiation and propagation [123]. The thermal properties of these filaments, including their glass transition temperature (Tg) and heat deflection temperature (HDT), impact their performance in high-temperature environments. These properties can vary based on the polymer matrix [124,125]. Fig. 9 displays the printed PLA and henequen fiber reinforced PLA composite specimens' differential scanning calorimetry (DSC) heating curves. The broad, low-intensity exothermic transition linked to the cold crystallization of a semi-crystalline polymer is represented by the first peak, which appears at 120°C. Furthermore, this peak indicates that a reorganization of the amorphous domains may occur during the heating of PLA and henequen fiber reinforced PLA composite specimens, thereby promoting the formation of crystalline structure. The melting of the crystalline structures created during the cooling of 3D printed samples and the cold crystallization are indicated by the second peak, which occurs at higher temperatures (∼150 C) and is associated with an exothermic transition.

**Fig. 10:**
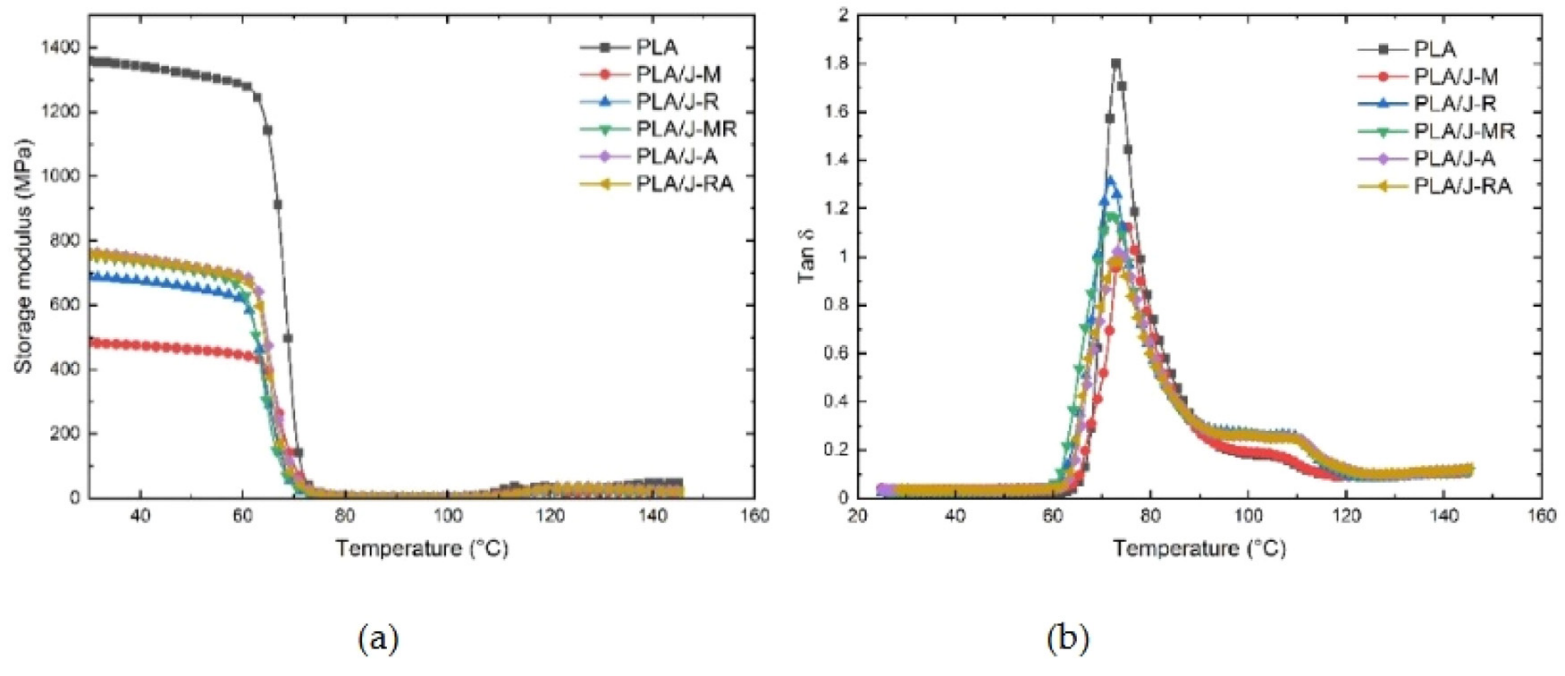
DMA curves between PLA and PLA jute fiber composites [127].

Natural fiber reinforced filaments are lightweight because their density is usually lower than that of traditional composites. For applications where weight loss is crucial, this is advantageous. One important component of the material response of natural fiber 3D printing filament is its viscoelastic behavior. These filaments can deform under stress and return to their original shape because they have both elastic and viscous properties. During the 3D printing process, natural fiber filaments' viscoelasticity affects their capacity to take in energy, withstand deformation, and recover from mechanical loads. The Dynamic Mechanical Analysis (DMA) curves between PLA and PLA jute fiber composites are depicted in Fig. 10. According to all materials, the storage modulus essentially stays constant up until the point at which the rubbery plateau and the leathery region begin, which is indicated by the onset temperature, at which point the modulus abruptly drops. The cold crystallization of PLA's amorphous portion is linked to an increase in the storage modulus above 115 C. Some PLA jute fiber composites and PLA had comparable Tonsets.

Some natural fiber reinforced filaments exhibit good damping capacity, meaning they can absorb and dissipate vibrational energy. This property can be valuable in applications where vibration control is needed [128,129].

It is noteworthy that there can be substantial variations in the mechanical properties of natural fibre reinforced filaments depending on various factors, including the type of natural fiber utilized, the polymer matrix, the orientation of the fiber, and the processing conditions. To achieve the required mechanical properties for a particular 3D printing application, it is therefore essential to choose the right combination of materials and processing parameters.

### Microscopic analysis of 3D printed natural fiber filaments

**Fig. 11:**
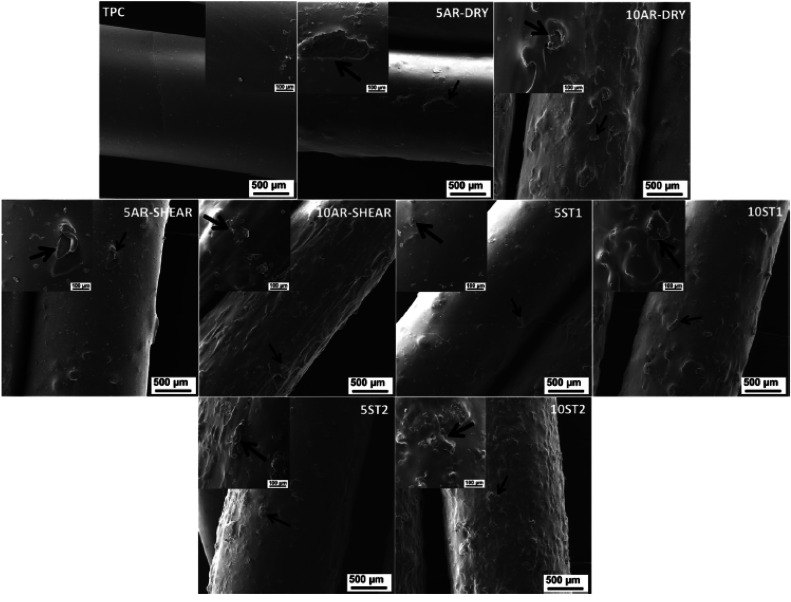
SEM images of thermoplastic copolyester filaments and soybean hull fibers mixed with thermoplastic copolyester composite filaments [130].

The microstructure and mechanical characteristics of 3D printed natural fiber filaments can be greatly understood by analyzing the material using microscopic Scanning Electron Microscopy (SEM). SEM provides a clear picture of fiber adhesion, dispersion, and the distribution of voids or defects at the micro scale, allowing for a thorough analysis of the interface between natural fibers and the polymer matrix. Understanding how the filaments' internal structure affects their mechanical strength and how they react to stress, deformation, and environmental conditions is made easier with the help of this analysis. In order to ensure that the micro scale characteristics of 3D printed objects using natural fiber filaments match macroscopic expectations, researchers and manufacturers using SEM imaging play a crucial role in optimizing material formulations. SEM pictures of soybean hull fibers combined with thermoplastic copolyester composite filaments and thermoplastic copolyester filaments are displayed in Fig. 11. In contrast to soybean hull fibers combined with thermoplastic copolyester composite filaments, the results demonstrated the smooth surface morphology of thermoplastic copolyester filaments. Some of the hulls of the composite filaments were exposed to the filament surface, giving them a rough surface morphology.

## Applications and industrial case studies

The use of natural fiber reinforced filaments in 3D printing offers a variety of existing and prospective uses in numerous industries. These filaments combine the adaptability of 3D printing technology with the advantages of sustainable natural fibers. Filaments reinforced with natural fibers are frequently utilized in product development and fast prototyping processes. They enable the development of working prototypes and the testing of designs by engineers and designers prior to final production. These filaments are used by sculptors and artists to produce elaborate and long-lasting works of art. Artistic projects gain visual appeal when they can be printed with natural colors and textures. Reinforced filaments made of natural fibers are used in educational settings to teach sustainability and 3D printing concepts. They give students opportunities for practical learning. These filaments are used by designers and enthusiasts to create personalized furniture, home accents, and architectural models. The fibers’ inherent beauty can improve interior design. Using natural fiber reinforced filaments, businesses investigating environmentally friendly packaging solutions can produce eco-friendly packaging material prototypes and samples [131,132]. Low-cost agricultural tools and equipment can be produced through 3D printing using natural fiber filaments in areas where access to conventional tools is restricted. In non-structural automotive components, natural fiber reinforced filaments may eventually take the place of conventional plastics, lowering weight and enhancing sustainability. The development of natural fiber-reinforced composites for use in aircraft, including lightweight structures and interior cabin components, is still under research [133,134]. For a visually appealing and environmentally friendly substitute, natural fiber reinforced filaments could be used in common consumer goods like kitchenware, smartphone cases, and sunglasses frames. Natural fiber filaments may be used in the production of customized medical devices, such as prosthetic limbs and orthopedic braces. Natural fiber reinforced filaments could be used to print architectural elements like decorative facades and eco-friendly bricks. Natural fiber filaments could be utilized to 3D print parts for sensors and equipment used for environmental monitoring [135,136]. In order to combine comfort and sustainability, the footwear industry should investigate the use of natural fiber reinforced filaments for specialized insoles and shoe components. The use of natural fiber reinforced composites in marine applications—such as non-structural boat components and interiors—is currently being investigated. These filaments could be used to make orthodontic devices and custom dental implants. Natural fiber reinforced filaments may find use in 3D printed solar panel components and wind turbine blades, supporting sustainable energy sources [[137], [138], [139], [140], [141], [142], [143]]. Though their full potential has not yet been reached, natural fiber reinforced filaments are already having an impact in some industries. Future developments in material science, 3D printing technology, and research and development will probably lead to the creation of novel and creative uses. These uses are in line with the expanding need in a number of industries for environmentally friendly and sustainable solutions [[144], [145], [146]].

### Case studies from industries

#### Automotive industry - ford motor company's sustainable dashboard components

Ford Motor Company has developed prototypes of dashboard panels using flax and hemp fiber-reinforced PLA filaments through FDM (Fused Deposition Modeling) 3D printing. In collaboration with suppliers like Magna and BASF, these bio-composite dashboards demonstrated a 25% weight reduction compared to traditional ABS plastic dashboards, contributing to better fuel efficiency. Ford also tested door panels and engine covers using similar composites, reinforcing their commitment to circular economy principles and CO₂ reduction.

#### Aerospace industry - Boeing's 3D printed cabin components

Boeing, in its initiative for lightweighting, has explored jute and flax fiber-reinforced composites for use in overhead bin lids, tray tables, and seat pan structures. These components, though non-load-bearing, require strict adherence to flammability and smoke toxicity standards. Boeing partnered with academic institutes to validate the thermal and mechanical stability of such natural composites in pressurized environments. This reduces the aircraft’s overall weight by 15-20 kg, translating to significant fuel savings over time.

#### Construction industry - 3D printed sustainable bricks for affordable housing

A team from ETH Zurich and the Indian Institute of Technology has developed interlocking 3D-printed bricks using rice husk and coconut coir reinforced PLA and clay composites. These bricks demonstrated compressive strengths of 4-6 MPa, suitable for low-cost housing. Unlike fired clay bricks, these are low-carbon, can be printed on-site, and reduce water usage. A pilot project in rural India used this technique to construct a small shelter in under 24 hours, demonstrating scalability and speed.

#### Consumer goods - custom 3D printed sunglasses

Eyewear startup W.R. Lab uses bamboo fiber/PLA composite filaments to create customized, lightweight sunglasses via desktop 3D printers. Each frame is tailored to customer specifications using scanned facial geometry, ensuring ergonomic fit. The composite not only offers aesthetic texture and rigidity but is also biodegradable. Tensile strength reached 50-60 MPa, and the printed sunglasses passed drop and torsion tests, making them a functional, sustainable consumer product.

The potential and adaptability of natural fiber reinforced filaments across various industries are demonstrated by these case studies. Businesses are lowering their environmental effect while simultaneously producing useful and creative products by utilizing the advantages of 3D printing technology and sustainable materials. We should anticipate more natural fiber filament integration into a wider range of industrial applications as research and technology progress.

## Conclusion

This comprehensive overview covers natural fiber filaments for 3D printing, including fabrication, characteristics, applications, and sustainability. Key findings are listed below:

• Pelletizing loose natural fibers into homogeneous pellets improves handling, storage, moisture control, and fiber-matrix bonding for smoother filament extrusion and fewer print errors.
• Flax, hemp, bamboo, and jute are perfect for sustainable 3D printing due to their mechanical qualities, biodegradability, and availability.
• Extrusion and its variants fabricate filaments, whereas fiber alignment, chemical surface treatments, and hybrid reinforcement improve mechanical performance.
• Natural fiber filaments provide exceptional strength-to-weight ratios and tunable thermal and morphological properties for automotive, aerospace, medical, educational, and consumer product applications.
• Lower carbon emissions, biodegradability, and renewable sourcing can help create a circular economy through recycling and upcycling.
• Moisture sensitivity, material inconsistency, scalability, and regulations are issues.
• Opportunities exist in innovative material formulations, smart materials, circular production, and industry adoption of sustainable alternatives.

## Ethics statements

The paper reflects the authors' own research and analysis in a truthful and complete manner.

## CRediT authorship contribution statement

**Senthil Maharaj Kennedy:** Conceptualization, Methodology, Software, Writing – original draft. **Lenin Anselm Wilson:** Conceptualization, Investigation. **Joemax Agu M:** Visualization, Data curation, Investigation. **Rajeev D:** Data curation, Investigation. **Jeen Robert RB:** Supervision, Writing – review & editing. **Balamurugan S:** Methodology, Software.

## Declaration of competing interest

The authors declare that they have no known competing financial interests or personal relationships that could have appeared to influence the work reported in this paper.
